# Easier Said than Done: The Politics of Medical Integration

**DOI:** 10.1007/s41649-024-00341-1

**Published:** 2025-05-09

**Authors:** Kathryn Muyskens

**Affiliations:** Centre for Biomedical Ethics, Yong Loo Lin School of Medicine, National University of Singapore, Singapore

**Keywords:** Integrative medicine, Healthcare justice, Traditional medicine, Indigenous knowledge

## Abstract

The WHO has recently been advocating for a more inclusive approach within healthcare systems, urging the integration of traditional medicines with biomedicine. Which traditions are perceived as authentic, authoritative or legitimate is as much a political question as it is a conceptual or scientific one. This paper will pose the question: is integration in fact desirable? Proponents may argue that integration will produce a number of benefits: more efficient use of healthcare resources, greater range of patient choice, and respect for the intellectual contributions of traditional medicines. Yet, I will argue that given the deep ontological divides across traditions, traditional medical practices are often incommensurable with biomedical paradigms, meaning integration is ultimately infeasible. Furthermore, to the extent that integration is pursued, it is likely to replicate colonial attitudes toward indigenous practices and repeat rather than remedy the very forms of epistemic injustice it would claim to avoid.

## Introduction

The concept of traditional medicines has been around since at least the end of the Cold War (where it emerged as a category as part of the decolonial movement led by many African countries) and engagement with traditional medicines by global institutions, such as the World Health Organization (WHO) has existed for nearly as long (see Ashworth and Cloatre 2022; Tilley 2021). Yet, in recent years, there has been increased attention placed on this category of medicine. In 2019, the WHO released a statement advocating for a more inclusive approach within healthcare systems, urging the integration of traditional medicines with biomedicine (WHO 2019). More recently, in 2023, the WHO held its first Global Summit on Traditional Medicine in Gandhinagar, Gujarat, India (WHO 2023). This move seems to be motivated by good intentions, like the wish to recognise the intellectual contributions of indigenous communities to medicine, as well as the wish to capitalize on local traditional knowledge and herbs for both economic and health gains. There are economic incentives as well. Indeed, the global market for herbal medicines is predicted to grow from $169.1 billion in 2023 to $279.8 billion by the end of 2028 (BBC Research 2023). For some, this move is a welcome one — a signal of a possible sea change in the way that traditional medicines had previously been regarded by many in the West. Yet, regardless of the win this may represent in terms of the perceived legitimacy of traditional medical knowledge and communities, this push for integration raises some serious challenges, both practical and moral.

Everyone needs medicine at some point in their lives. Yet the boundaries of who gets to fulfill that role and how are often contentious. Modern Western scientific medicine, often called biomedicine, is a relative newcomer to the milieu of medical traditions. Even so, it is this style of medicine which has gained mainstream and authoritative status — especially in the wake of WWII. Which traditions are perceived as authentic, authoritative or legitimate is as much a political question as it is a conceptual or scientific one. As Coderey and Pordie (2022) write in the introduction to their edited volume, *Circulation and Governance of Asian Medicine*, integration of biomedicine and traditional medicines in Asia has often been initiated by states, who see in this integration the potential to extend health coverage in both demographic and financial terms. Additionally, in contexts like India, Vietnam and Myanmar, integration of traditional medicines has been seen as part of the post-colonial nation-building process, while in other countries (like China, see Chee 2021 and Brazelton 2019) it was part of a nation development-modernization program (Coderey and Pordie 2022). In either case, however, the motivation was often tied to a strong nationalistic ethos, and a desire to preserve culture and identity, while resisting the growing dominance of the West (Coderey and Pordie 2022).

This paper will explore a more radical take on this issue, posing the question: is integration in fact desirable? In recent years, the assumption seems to have been ‘yes,’ with advocates for diverse medical traditions pushing for increased recognition and integration with biomedicine (Park and Canaway 2019). Proponents argue that integration will produce a number of benefits, some practical (e.g. more efficient use of healthcare resources and increases in patient wellbeing) and some moral (e.g. respect for patient choice, respect for the intellectual contributions of traditional medicines, etc.). Yet, in this paper, I will argue that integration across medical paradigms is unlikely to lead to these outcomes. In fact, integration (in the way that it is typically practiced) runs against the interests of patients, practitioners, and the heritage traditions themselves. To make my case, I will recount some of the political dimensions that shape the traditional medical landscape, and sketch some of the roadblocks (both ethical and practical) that the integrative approach faces. I will demonstrate that given the deep ontological divides that separate many traditional medicines from biomedicine, integration is ultimately infeasible. Furthermore, to the extent that integration is pursued, it is likely to replicate colonial attitudes toward traditional medical and indigenous practices and repeat rather than remedy the very forms of epistemic injustice it would claim to avoid.

## “Traditional” Medicines and “True” Scotsman

Whenever discussing the medical landscape, terminology is both important and often highly problematic. In lay terms, the word “medicine” in its unqualified and unmodified usage is assumed to refer to the Western scientific medical practice or biomedicine. In light of biomedicine’s dominant status, other styles of medicine have been relegated to the side lines and acquired linguistic qualifiers such as “traditional,” “ethnic,” “vernacular,” “alternative” and “complementary.” These labels have always attracted controversy. On the one hand, they are criticized for expressing a colonial and ethnocentric attitude towards medicines from non-Western cultures (see, Morreim 2003; Kirmayer 2011; Kidd 2013; Muyskens 2022, 2024), and on the other, some hard-line skeptics have argued that none of these categories deserve to be included in the medical family to begin with, and should instead be seen as “alternatives to medicine” (see, Pigliuchi and Boudry 2013; Schneiderman 2000; Torcello 2013; Shahvisi 2016). Often, the attempts to label and categorize medical traditions obscure as much as they illuminate. There are large catch-all terms, like “CAM” (Complementary and Alternative Medicines) and “T&CM” (Traditional and Complementary Medicines) — which may include anything from new age medicines like crystal therapy to ancient practices like Ayurveda. Still, more precise distinctions are often contentious as well. How old must a medical system be in order to be “traditional,^1^” for example? What makes a medicine “ethnic”? What does it mean to be “alternative”?

The application of such terms often resembles the ‘No True Scotsmen’ fallacy, leaving the category slippery and very nearly meaningless (possibly by design). Among logicians, the ‘No True Scotsman’ fallacy is also known as the “appeal to purity.” It is a type of informal logical fallacy. The speaker asserts a generality, like “no Scotsman puts sugar on his porridge” or “traditional medicines are harmless.” Then, when presented with evidence to the contrary (for example, your uncle Angus, who is Scottish — and definitely puts sugar in his porridge, or for another example, you might observe that some traditional remedies contain adulterated and poisonous substances that would certainly be harmful to ingest), the speaker replies dismissively by invalidating the example as unrepresentative (i.e. Angus isn’t a “true scotsman” or the particular remedy in question “isn’t the *real/authentic* tradition”).

The ‘No True Scotsman’ fallacy uses emotionally charged, vague language to evade criticism. But it ultimately leaves the objection unanswered. Who really gets to say what counts as a “True” Scotsman? Or a “traditional” medicine? The legitimacy of a particular medical tradition’s categorization is far from a simple scientific question, and is often tied to its cultural and historical context, as well as to the power dynamics at play in society. We need precise terms in order to meaningfully discuss the merits of each practice. Fuzzy terms like “traditional medicine” invite opportunists and motivated reasoning, but better terms are always available. Thus, for the purposes of this paper, I will be forced to adopt the existing language.

In my other work, I have argued for an inclusive approach to what counts as a medical tradition (see Muyskens 2023). To navigate around the inherent difficulties with these labels, I have offered a culturally pluralistic account of medicine wherein medicine is defined first and foremost as a relationship with several necessary parts: a patient, a practitioner, a theoretical framework, and a set of treatments or remedies. Under this definition, practices like Yunani, TCM, and biomedicine are all equally worthy of being called medical. No caveats or modifiers are necessary. Even so, sometimes the distinction between medicines which claim to be “traditional” and other practices which merely claim to be “alternative” or “complementary” can be important. This distinction is most relevant in contexts where the history of colonialism has shaped the trajectory of medical practice and the status of a particular medical tradition.

While I will try to be sensitive to this history and its implications for epistemic injustice (discussed in more depth below), it is also important to acknowledge the political power of the “traditional” label. That said, in what follows, I will use the language each medical tradition uses to describe itself. Traditional Chinese Medicine (TCM), for instance, calls itself traditional. So, I will also refer to it as such. But for the arguments that follow, it is important to keep in mind that these terms are always contentious. Traditional medicines are often not as traditional as they claim, and indeed, for that matter scientific medicine is often not as scientific as it would claim to be either. Writing about the “scientificness” of scientific medicine, Morreim (2003, 222) puts it,

Standard medicine is not nearly so scientific as is usually assumed. Among other factors, there are far too many phenomena to study; limited research resources are often directed as much by political and commercial interests as by medical needs; actual practices do not reflect well the science that has been gathered; the most pristine science is often the least useful in the real-world care of ordinary patients.

Similarly, with regard to traditional medicines, the truth of just how traditional a practice is may be difficult to determine. For some prominent examples, we can look at TCM and yoga. TCM indeed has deep roots, with at least one of its founding texts (the *Huangdi Neijing*) dating back to as early as the second century CE. Yet, the form of TCM that most people practice today in fact only dates back to 1950s China, when Chairman Mao standardized the practice (in part in a bid to use it as a form of international soft-power) (Levinovitz 2013). Likewise, the first mention of yoga in the Hindu texts dates back to the Katha Upanishad, which is estimated to be written in the fifth or sixth century BCE. Even so, the type of yoga that most people now practice (with its focus on asanas and the familiar ‘sun salutation’) bears little resemblance to earlier forms, and has been heavily influenced by the wellness and fitness industries since the 1920s (Singleton 2010).

Thus, when asked which is the *real* traditional medicine, there can be no answer. Firstly, the question is embedded with inaccurate presumptions — there is no one true tradition to be found. Authenticity and authority within a tradition is essentially and continually constructed (and reconstructed) as the situation demands. Secondly, (as I will explain further in the next sections) the question can be a dangerous one to answer — inevitably pitting one community against others. To answer this loaded question is to establish a pole, from which a perimeter can be set, ultimately consigning some parts to the fringes and thereby delegitimize and disenfranchise them.

## To Integrate or Not to Integrate? That is the Question

Despite this lack of linguistic or conceptual clarity, the WHO and many healthcare systems seem to be moving more and more to embrace integrative medicine, and indeed this trend has been gaining momentum for some years. Even back in 2001, some authors were writing optimistically about it. For example, Kaptchuk and Eisenberg (2001) note that CAM medicines have begun to be recognised as “cohabitants” in a Postmodern medical network in which consumer preferences dictate the services offered. But, all this hype has overlooked the more basic question: is integrative medicine (defined as the practice of combining various medical traditions in one healthcare system) a desirable thing to do? As I have indicated in the introduction, I have my doubts that it is. However, before I get to my reasons for thinking so, I will do my best to explain the case in favor of integration to the best of my ability. This is, of course, easier said than done, since what integration looks like in practice, varies widely depending on the traditions involved and the existing healthcare policy landscape. Integration might be taken to mean that multiple modalities are available within one hospital. For example, Singapore's health policy has embraced integration in some hospitals, and some offer both acupuncture and hypnotherapy along with conventional biomedicine on the same premises (Low 2013). Integrative medicine can also take place at the system level, e.g. different practices share the same network in terms of insurance coverage and/or referrals, but need not be housed in the same locale.

In its ideal form, integrative medicine seeks to combine Western biomedicine with traditional, complementary and alternative therapies for a more holistic healthcare system. Proponents argue that this can increase patient autonomy (and by extension patient satisfaction), since patients can choose from a wider range of treatments and therapies and tailor them to their needs and preferences. At least in theory, integrative medicine can also allow for better coordination across treatments and methodologies to potentially reduce side effects. For instance, patients can minimize the side effects of chemotherapy by incorporating acupuncture or massage (Li et al. 2019). Additionally, integration allows for a more holistic health experience, since many traditional and complementary medical practices combine attention to the body with attention to the mind and spirit, which has been observed to lead to improved outcomes (Bahall 2017). Lastly, integrating traditional practices may offer alternative treatments for conditions that are not well-addressed by biomedicine, for example, patients with many chronic conditions like fibromyalgia, migraines, and chronic fatigue syndrome (and other conditions primarily characterized by chronic pain) have been observed to find relief through TCM (Fisher et al. 2016; Bahall 2017; Vickers et al. 2012). Pragmatically speaking too, practitioners of traditional and complementary medicines could also stand to benefit from integration. Though it is worth noting that in practice many practitioners of traditional and complementary medicines are skeptical about integration for their own reasons, at least in theory, the integrative approach *could* translate to an increase in perceived legitimacy, and perhaps also a new influx of patients (if conventional biomedical doctors were to connect their patients with other medical practitioners through referrals). Thus, integrative medicine is said to be good for patients (for their autonomy and wellbeing), good for practitioners (in terms of cultural respect and access to patients), and good for society (in the sense of reducing ethnocentric bias and epistemic injustices).

If true, these are all genuine and important gains to be made. But as I will show in what follows, what is good in theory is sometimes bad in practice. Without attention to the practical barriers to effective integration, the policy will not be able to deliver on its promises. Done carelessly, in fact, integrative medicine has the potential to produce exactly the opposite results – to worsen patient health, injure public trust in medicine and science, and even contribute to worsening epistemic injustice and inflict its own kind of cultural imperialism on traditional medical communities. In what follows, I will demonstrate how the deep ontological fissures in the conception of medicine across divergent paradigms undermine the practice of integrative medicine.

## The Case Against Integration

To reiterate, integrative medicine is purported to benefit both patients and practitioners, and to align with the interests of social justice by rectifying past instances of epistemic injustice or colonial domination. If true, these are all worthy goals. Yet, as I will demonstrate, integrative medicine (at least as it is practiced) is unable to deliver on these points. To make my case, I will focus on the example of TCM and biomedicine. Three major roadblocks are worthy of focus, the ontological problem of incommensurable paradigms and the twin ethical problems of epistemic injustice and cultural imperialism.

### Incommensurable Paradigms

Chief among the barriers to integration is the fact that traditional medicines, like TCM, operate under completely different conceptual frameworks compared to biomedicine. These different conceptions of health and medicine are not reconcilable with one another. For instance, in TCM the boundaries and functions of “Spleen Qi” are not identical to the boundaries of the literal organ as biomedicine would describe it. TCM and biomedicine also differ in their aims and scope. Western medicine is known for its focus on pathology, and it has made great strides in the treatment of acute and infectious illness over the centuries. TCM, in contrast, often aims at longevity and wellbeing. Thus, while both traditions of medicine aim at human health, the different ontological commitments and metaphysical assumptions shape the practice in important ways. Western medicine aims to alleviate pain and dysfunction. TCM aims to restore the balance of qi, within the person and between the person and their environment. Naturally, along with those differences in orientation come different views of what kinds of behaviors come under the purview of the medical.

These kinds of differences make each of these medical traditions fundamentally incommensurable with one another. I borrow this notion of incommensurability of paradigms from the terminology utilized by Kuhn (1970, 1982) in his work in the philosophy of science. When two paradigms are incommensurable, even if they use superficially similar language (e.g. the use of “element” or “atom” in both Ancient Greek and modern physics, or “wellbeing” in TCM and biomedicine, etc.), it is ultimately impossible to define the terms of one theory in the vocabulary of the other (Kuhn 1982). Indeed, this is exactly what we find when we look closely at something like TCM in comparison with biomedicine. Practitioners of both may speak of patient health and wellbeing, but the concepts within each tradition encompass different phenomena (e.g. qi and meridians have biomedical counterparts). This incommensurability is the root of much of the problems with any attempt to integrate these two medical traditions. The standards of one cannot be appropriately applied to measure the other (though not for lack of trying). This makes communication between biomedical and traditional practitioners cumbersome, and coordination of care complicated, if not impossible. Failing to recognize this can have serious consequences for patients, practitioners, and the traditional medicines themselves.

Though some scholars are skeptical about how deep this ontological divide between “orthodox” and “unorthodox” medicines truly runs (see Han 2002) I take this incommensurability to be an unavoidable fact. Even so, in practice, it is often ignored or minimized. When integrating divergent traditions of medicine, often the context of a remedy is lost — and this can lead to many negative outcomes, including harm to the patient. For one example, we can look at “dry needling” — a practice used in physiotherapy, that was co-opted from acupuncture, but is now applied without reference to the principles of TCM (Fan and He 2015). Physiotherapists may thus apply the treatment in ways TCM practitioners would not recommend, including using acupuncture points that would be contraindicated on pregnant women because of a risk of miscarriage (McDowell et al. 2019). Since the physiotherapist’s ontological paradigm does not include concepts like qi or meridians, this hinders any kind of productive exchange between TCM practitioners and physiotherapists regarding patient wellbeing and treatment. At minimum, in an integrative medicine setting, this ontological divide undermines collaboration and can inflame the potential for epistemic injustice (see below).

This problem is far from limited to interactions between TCM and biomedicine, extending to other traditions and to other areas of science. Indeed, as Ludwig (2016) has written, speaking about the case of indigenous Australian communities, despite the increasing interest in ‘taking indigenous knowledge seriously,’ recognition typically extends only insofar as it is useful for Western science. This asymmetrical engagement undercuts the sincerity of any real exchange of knowledge.

### Epistemic Injustice

In addition to the incommensurability problem, there is also a moral hazard associated with integrative medicine regarding epistemic injustice. The term epistemic injustice was first coined by Fricker (2007), as a way of articulating the ethical harms associated with failure to recognize individuals and communities as legitimate sources of knowledge. The classic example is the case of testimonial injustice and sexist biases, for example, women’s experiences and testimony being taken less seriously than those of men (Fricker 2007).

We can find a similar discounting of knowledge in the relationship between medical paradigms. Many practitioners of traditional medicines are minorities, if not always ethnically, at least professionally within the medical scene. For example, TCM’s link to Chinese culture makes it part of the majority in one sense within Singapore, but still a minority when it comes to the medical profession, since as of 2021 there were only 3320 TCM practitioners compared to 15,000 doctors (Goh 2022). In the medical and research marketplace, TCM is often discounted and derided as unscientific or lacking evidential support. Yet, as I have laid out in the previous section, it may not be appropriate to apply the standards of the biomedical paradigm to TCM in the first place, due to the underlying ontological differences. Though, because of the current power dynamics, this does not prevent many TCM practitioners and researchers from trying to justify their practices in modern scientific language (to limited effect). Further integration into the mainstream healthcare system can be attractive for financial reasons for many practitioners. In theory at least, integration can provide greater access to patients, connecting practitioners with wider patient populations who they otherwise may never have encountered. It is also often assumed that integration into mainstream healthcare will mean an increase in perceived credibility. These are powerful incentives, but doomed to frustration given the divergent ontological commitments.

It is important to note that even when well intentioned, integrating such divergent styles of medicine is hardly an egalitarian practice. In practice, biomedicine is the dominant style in most healthcare systems across the globe, and thereby integration in large part means that traditional medicines, like TCM, must compromise and make themselves legible to biomedicine before they can be assimilated into the mainstream structure. As Park and Canaway (2019) discuss, integration of biomedicine with traditional and complementary medicines is touted to contribute to the improvement of healthcare quality through increased regulation and standardization of traditional medicines, and to improve equitable access to healthcare by expanding insurance coverage for traditional medicines, as well as improving accountability and monitoring. Notably, the rhetorical tilt of their article, and others like it, underscores the idea that traditional medicines will be “brought into alignment” or “brought up to standard” with biomedicine. It is not framed as a mutual exchange. For traditional practitioners and communities who have concerns about preserving the cultural heritage and authenticity of their practices, integrating with Western biomedicine could do more damage than good, because the process inevitably compromises the traditional nature of their treatments. As further evidence of this, we can look at the case of Tibetan traditional medicine as described by Vincanne Adams in the article, “Randomized Controlled Crimes: Postcolonial Sciences in Alternative Medicine Research” (2002). Adams (2002) describes how the prioritization of randomized controlled trials and the quest for ‘active ingredients’ created tension between what gets counted as ‘medical fact’ as opposed to mere ‘medical belief,’ and this tension has led not just to the marginalization of traditional practices but to their outright criminalization as well. Thus, where integrative medicine has the consequence of standardizing and formalizing traditional remedies, this too, I think, is a double-edged sword. The impetus for such standardization is often rooted in good intentions, like minimizing the risk to patients in the case of adulterated substances in herbal remedies, for example. Yet, it also raises the question of *who* gets to decide what the standard is and *how* to implement the process of formalization.

This is a concern that extends beyond TCM to other traditional practices as well. Many patients across the world rely on traditional medicines as part of their cultural heritage, and its philosophies shape the way they conceive of their own health. In fact, according to Lee et al. (2022), global use of traditional, complementary and alternative medicines “was found to be substantial, ranging from 24 to 71.3% in different countries” (713). Ensuring that traditional practices are not co-opted or commodified by biomedicine is particularly important given the history of colonialism and exploitation of non-Western medical practices (see Cloatre 2019; Das 2019 for more on this history).

There are many negative consequences to forcing traditional practices to conform to biomedical standards. This includes an increased burden on practitioners to become literate in multiple medical paradigms — which can be both financially costly, time consuming, and cognitively challenging. This is true no matter what side of the integrative relationship the practitioner is on — whether biomedical physicians or acupuncturists. Because medical resources are almost always in short supply, these increased costs are worth taking seriously. Healthcare costs may go up in hidden ways as well, as doctor’s must spend more time in training, and this may make care less abundant and harder to access. Neither outcome is in the interest of healthcare justice.

Here, some may wonder whether the threat to cultural heritage when it comes to medical traditions is overblown. After all, it is true that medical traditions do not have the same degree of ‘unchosen-ness’ as other aspects of cultural identity. People are free to engage with any medical tradition they choose, and many are quite happy to be what I have called “medical omnivores” (Muyskens 2023). If the analogy between respect for different cultural identities and respect for different medical traditions is undermined, then the ethical case against integration (and its tendency to place pressure on traditional medicines to conform to biomedical norms) would be undermined.

Yet, as I have argued elsewhere, “[w]hat is at issue here, however, is not which medicines people actually choose—or who chooses what medicine. Rather, it is a question of what people are able to choose and what options they are given in the first place” (Muyskens 2024, 9). To clarify further, the ethical problem here is not merely that traditional medicines alter their practices. All medical practices naturally evolve to one degree or another over time as more is learned about human health and new health conditions arise. The ethical issue here is one of coercion to evolve in a particular manner — specifically, to conform to the style of practice popular in biomedicine. In the case of many integrative models, there is often no realistic option for the traditional medical practitioners to direct that change as they see fit — rather, the change comes from the pressure to conform to the assumptions of the dominant paradigm. Sometimes this change may be beneficial to patients (e.g. when the imposition of standardization and regulation of herbal remedies ensures that these do not contain adulterated or toxic substances), but at other times the change is less justified. Demanding that a traditional medicine meet safety standards with regard to its herbal preparations might be coercive, but the coercion is justified by the need to protect patients. But other impositions on the format and delivery of traditional practices, especially with regard to the causal explanations behind the traditional remedies, are not justifiable in the same way. Asking a tradition like TCM to explain its remedies in scientific language in order to be taken seriously is the equivalent of insisting that all medicine be conducted in English or Latin. Forcing qi into a scientific category is both nonsensical and unnecessary. This type of integrative medicine can represent a coercive pressure on traditional practices, and where these practices are deeply valued by indigenous or minority communities, this might rise to the level of cultural imperialism — which I will discuss in the following section.

### Cultural Imperialism and (Un)intentional Coercion in Medically Plural Contexts

If integrative medicine is to deliver on its claims of social justice, the integration must be done in a way that respects the cultural and historical context of those practices and ensures that they remain under the control of the communities that developed them, otherwise, integration becomes cultural imperialism by another name. The political philosopher, Fuller (2012), describes the hazard of cultural imperialism in the related context of health humanitarianism carried out by International Non-Governmental Organizations (INGOs). She explains that even without intending to do so, health INGOs can be guilty of coercion because of the structure of the offer of aid (Fuller 2012). As an example, she describes an INGO wishing to provide antiretrovirals to an HIV-affected community. Conflict arises from the fact that the antiretrovirals are known to negatively interact with local herbal remedies provided by a traditional healer (Fuller 2012, 216). She argues the INGO has a few options: (1) it can undermine the healer and convince the patients to stop taking the herbal remedies, (2) the INGO could relocate to serve a different community, or (3) the INGO could attempt to convince the traditional healers of the negative interaction between the antiretrovirals and traditional remedies (Fuller 2012, 232).

Fuller argues that the first two options are culturally imperialistic since they send the message that the recipient community must either conform to the INGO’s values or go without help. The third option is the only option which she thinks avoids imperialistic harm (Fuller 2012, 214). In the present context, a similar offer seems to be involved in the push for integrative medicine. On the surface, it seems like a move towards inclusion. Yet, in practice, the offer puts pressure on traditional medicines to conform to biomedical norms in order to survive at all.

We can examine how Fuller’s insights could shape the implementation of an integrative medicine policy regarding TCM and biomedicine. Having just recently introduced the new policy, *Healthier SG*, which aims at taking a more holistic view of healthcare, Singapore presents an excellent case to explore the implications of integration (Ministry of Health 2022). With *Healthier SG*, patients are meant to create long-term health-plans with the guidance of family doctors. Where does TCM fit into that picture? Officially, TCM is given a place in the *Healthier SG* plan (Ministry of Health 2022). On the surface, this means there is potential for more open collaboration and dialogue between conventional medical doctors and TCM practitioners regarding patient care planning. This, of course, relies on the patient’s willingness to disclose their use of herbal remedies to their biomedical doctors, and/or their use of pharmaceuticals to their traditional medical doctors. Yet, even in a society where both TCM and biomedicine are officially recognised and widely available, this disclosure is not always forthcoming. Approximately 45% of the population has used TCM or another traditional medicine in the last year, and yet most do not disclose this to biomedical professionals, for fear of being scolded (Chan 2001; Kelak et al. 2018).

When a biomedical doctor is faced with a patient who wishes to incorporate a traditional practice into their care, they will be faced with a similar puzzle to the INGO workers in Fuller’s example. They could advise their patient away from traditional remedies, in order to avoid what they see as ineffective or possibly dangerous potential interactions, or they could recommend the patient see a different physician — one with more knowledge of traditional practices. Both would again send the signal that the use of traditional medicines is a barrier to the patient’s access to care. But the third option, of actively collaborating with the traditional practitioner, is a burdensome and complicated task. If all parties are not sufficiently willing to meet in the middle and work toward a shared understanding in an equitable manner, the project of integration will naturally and inevitably devolve. Further, the problem of incommensurable paradigms makes successful communication and collaboration nearly impossible beyond a surface level. These roadblocks should not be ignored. It is not my argument in this paper that integration is inevitably problematic. Yet, until these roadblocks I have articulated are acknowledged and dealt with, integration is likely to create more problems than it solves.

## Conclusion

In conclusion, to the question of whether integration of traditional medical practices into mainstream biomedical care is desirable my answer is, no. The promises of the benefits, whether to patients, practitioners, or the traditional medicines themselves, are hollow. Ignoring or underestimating the very real challenges of trying to coordinate care across incommensurable medical paradigms will only lead to disaster. As I have demonstrated in this paper, integration in practice typically means a watering down of traditional medicine, a removal from its roots and cultural context, and a subjugation of its contributions as second class even as they are assimilated into the mainstream. Because of the incommensurable differences in the conception of medical practice and healing, integration cannot be managed on a truly level playing field, it cannot deliver on its promises — and may instead deliver exactly what the skeptics have feared. It could not only worsen the history of epistemic injustice done to traditional medical communities, but also make healthcare more costly, cumbersome, ineffective, and thereby injure public trust in the institution of medicine as a whole. That said, there is a viable alternative. A *pluralistic* landscape, where different styles and paradigms of medicine can be recognized and allowed to coexist in parallel with one another, would be preferable, where multiple traditions of medicine are legal and permitted, but not forced to assimilate into one (in)coherent whole. This kind of approach may have its own unique challenges, as some authors have already observed (see Wahlberg (2007) for one example) and more work on this area is certainly needed. Even so, the arguments made in this paper against the integrative approach still remain. Ultimately, the goal should be to provide the best possible healthcare for patients, while respecting the cultural and historical context of traditional medical practices. For all of these reasons, this will mean that integration should be avoided in favor of pluralistic policies instead.

## Data Availability

Not Applicable

## Footnotes

1 See Carlessi (2024) for an exploration on how the WHO has used the concept “traditional medicine” since the 1960s, and how it has transformed over time.
